# Translation of a Diabetes Remission Service into Australian Primary Care: Findings from the Evaluation of DiRECT-Australia

**DOI:** 10.1155/2024/2350551

**Published:** 2024-02-08

**Authors:** Ritesh Chimoriya, Kimberly Mitlehner, Chee L. Khoo, Uchechukwu Levi Osuagwu, Russell Thomson, Lei Si, Michael Lean, David Simmons, Milan K. Piya

**Affiliations:** ^1^School of Medicine, Western Sydney University, Campbelltown, NSW, Australia; ^2^Healthfocus Family Practice, Ingleburn, NSW, Australia; ^3^Bathurst Rural Clinical School (BRCS), Western Sydney University, Bathurst, NSW, Australia; ^4^School of Computer, Data and Mathematical Sciences, Western Sydney University, Penrith, Australia; ^5^School of Health Sciences, Western Sydney University, Campbelltown, Australia; ^6^School of Medicine, Dentistry and Nursing, College of Medical, Veterinary and Life Sciences, University of Glasgow, Glasgow, UK; ^7^Campbelltown and Camden Hospitals, Campbelltown, NSW, Australia

## Abstract

**Background:**

The Diabetes Remission Clinical Trial (DiRECT) study demonstrated that an intensive and structured weight management program in UK primary care resulted in high rates of diabetes remission in adults with recent onset type 2 diabetes mellitus (T2DM). This study was aimed at evaluating the translation of the DiRECT intervention into an Australian primary care setting.

**Methods:**

All patients enrolled in the DiRECT-Australia Type 2 Diabetes Remission Service in a region of Sydney (Macarthur region, South Western Sydney, Australia) were included. Eligible participants were aged 20–70 years, noninsulin treated, with T2DM of ≤6 years' duration, and body mass index (BMI) ≥ 27 kg/m^2^. Total diet replacement of 825-853 kcal/day using meal replacements was implemented for 12 weeks, followed by an ongoing structured program until 52 weeks, with regular follow-up with a general practitioner, dietitian, and/or practice nurse.

**Results:**

Of 39 recruited participants, 32 (82.1%) and 27 (69.2%) completed 12 weeks and 52 weeks of the structured program, respectively. Decrease in weight by -12.0 kg (95% CI: -9.6, -14.4; *p* < 0.001) and -9.1 kg (95% CI: -5.2, -12.9; *p* < 0.001) and decrease in glycated haemoglobin (HbA1c) by -1.1% (95% CI: -0.6, -1.6; *p* < 0.001) and -0.6% (95% CI: -0.1, -1.1; *p* = 0.013) were observed at 12 and 52 weeks, respectively. At the end of 12 and 52 weeks, 93.8% (30/32) and 55.6% (15/27) of those with follow-up data met the criteria for diabetes remission, respectively. Quality of life and wellbeing scores increased over the course of 12 weeks, remaining significantly higher at 52 weeks. Participants reported they would be willing to pay A$92.50 (95% CI: A$75.80, A$109.30) per fortnight for the low-calorie meal replacement shakes.

**Conclusions:**

These findings support the feasibility of a structured diabetes remission service in an Australian primary care setting to achieve improvements in glycaemia, weight, and quality of life and wellbeing, and suggest a substantial willingness to pay for diet replacement products among participants.

## 1. Introduction

Type 2 diabetes mellitus (T2DM) is a leading clinical and public health concern [1, 2], with its global prevalence estimated to rapidly increase in pandemic proportions from 10.5% in 2021 to 12.2% in 2045, and resulting in approximately 11.5% of the total global health expenditure in 2021 [3, 4]. In Australia, the prevalence of T2DM has been rising in parallel with the obesity epidemic, and an estimated 5% of the Australian adult population were reported to be living with T2DM in 2017-18, with many more undiagnosed [5]. T2DM has become one of the critical challenges currently confronting Australia's healthcare system, which is associated with over one million hospitalisations and an annual estimated A$3 billion expenditure in the Australian health system [5, 6].

Current guidelines for the management of T2DM recommend lifestyle modification through diet and physical activity as part of first-line therapy for patients with recently diagnosed T2DM [7–9]. Weight loss is an essential component of the management of T2DM, and individuals with T2DM who also have overweight or obesity are often recommended to achieve and maintain at least 5% weight loss due to its associated improvements in glycaemia [10–12]. Moreover, studies have shown that weight loss of at least 10 to 15 kg, achieved through lifestyle interventions, very low-calorie diet, or bariatric surgery, can result in normalisation of blood glucose concentrations in some individuals with short-duration T2DM [13–15]. As T2DM is strongly associated with weight gain and accumulation of excess fat within the liver and pancreas, it can potentially be reversed by treating the underlying process of body fat accumulation [16, 17]. Inducing substantial negative energy balance through dietary intervention (600–700 kcal/day diet) can lead to liver insulin resistance and fat content normalisation within 7 days and first-phase insulin response and pancreas fat content normalisation over 8 weeks [18, 19]. A recent international consensus report has also acknowledged the potential of remission in T2DM, and recommended remission to be defined as “a return of glycated haemoglobin (HbA1c) to <6.5% (<48 mmol/mol) that occurs spontaneously or following an intervention and that persists for at least 3 months in the absence of usual glucose-lowering pharmacotherapy” [20]. For lifestyle interventions, the consensus recommended that testing of HbA1c to document diabetes remission should be performed just prior to the intervention and at least 6 months after beginning the intervention and 3 months after cessation of any pharmacotherapy [20]. With the current guidelines recognising the potential for diabetes remission through substantial weight loss [7, 8, 10], the focus on the management of T2DM has shifted from an upward titration of medications to reversing the disease progression through early significant weight loss [21, 22].

Challenging the conventional view that T2DM is a permanent and progressive disease, the Diabetes Remission Clinical Trial (DiRECT) conducted in the UK demonstrated that an intensive and structured weight management program delivered in primary care can result in significant weight loss and diabetes remission among adults with recent onset T2DM [17, 23, 24]. In the DiRECT UK study, a quarter of participants lost at least 15 kg of body weight, and almost half of the participants achieved diabetes remission at 12 months. At the end of two years of the program, more than a third of the participants achieved sustained remission, and 70% of those who maintained a weight loss of more than 15 kg remained in remission [17, 23].

The DiRECT-Australia T2DM Remission Service was set up with the intention to replicate the DiRECT UK intervention [25] in an Australian primary care setting. However, the healthcare system in Australia differs markedly from that in the UK, with many single or small general practices, fewer practice nurses, and limited availability or access to allied health professionals in a multidisciplinary setting [26]. Australia also has a federal-state funding divide, with hospitals funded mainly by the states alongside a federally funded national fee-for-service remuneration system, which may impact the translation of the diabetes remission service approach into Australian primary care. Therefore, this study was aimed at evaluating the implementation of the DiRECT intervention in an Australian primary care setting. The primary research question was as follows: Can a structured and evidence-based T2DM remission service developed in the UK be successfully replicated in an Australian primary care setting to result in remission of diabetes at 12 and 52 weeks in patients with recent onset T2DM?

## 2. Materials and Methods

### 2.1. Study Design and Recruitment

This is a pilot prospective evaluation of the DiRECT-Australia T2DM Remission Service conducted between February 2019 and July 2022. A new service for the implementation of DiRECT T2DM Remission Service was set up in an Australian primary care setting in a region of Sydney (Macarthur region, South Western Sydney, Australia) based on the DiRECT UK model [25] in February 2019. All participants in the service were recruited by general practices in a region of Sydney, Australia. Following referral by the general practices, the patients were screened based on the eligibility criteria of the DiRECT UK [25] prior to their inclusion in the service. All patients enrolled in the DiRECT-Australia T2DM Remission Service were invited to participate in this evaluation study, and only those who consented were included in the study.

### 2.2. Inclusion and Exclusion Criteria

Eligible participants were aged 20–70 years, with T2DM of ≤6 years' duration, body mass index (BMI) ≥ 27 kg/m^2^, HbA1c ≥ 6.5% (≥48 mmol/mol) if only on diet-based intervention in the past 12 months, and HbA1c ≥ 6.0% (≥42 mmol/mol) if on treatment with noninsulin diabetes therapy. Exclusion criteria included current insulin use, recent HbA1c ≥ 12% (≥108 mmol/mol), weight loss of more than 5 kg in the past 6 months, or a diagnosis of cancer, severe heart failure, myocardial infarction, or any major mental health issues. Pregnant women or those considering pregnancy, people with cognitive impairment, and those currently participating in another weight loss program were also excluded.

### 2.3. The DiRECT-Australia Intervention

The DiRECT intervention was delivered in three phases based on the DiRECT UK protocol [25]. The intervention was embedded within routine care, with additional dietitian time provided by the DiRECT-Australia T2DM Remission Service. Regular follow-up was with a member of the clinical team which included a general practitioner, dietitian, and/or practice nurse. The participants were also provided with a printed booklet containing detailed information on each phase. The three phases of the DiRECT intervention are detailed below.

#### 2.3.1. Phase 1: Total Diet Replacement (Weeks 0-12)

All oral antihyperglycaemic agents and antihypertensive medications were withdrawn, and a total dietary replacement with liquid formula diet (825-853 kcal per day; 80-108 gm/day carbohydrate, 10-14 gm/day fat, 64-84 gm/day protein, and 10-12 gm/day fibre) in the form of four meal replacement shakes/soups per day was implemented for 12 weeks. These shakes/soups were sourced from the same company that supplied the DiRECT study in the UK (Counterweight UK, London, United Kingdom). Participants were advised to drink over 2.25 litres of water per day in addition to the fibre supplement, as required. Participants were clinically evaluated one week after the commencement of the total diet replacement and then on a fortnightly basis by a member of the clinical team.

#### 2.3.2. Phase 2: Food Reintroduction (Weeks 13-18)

The structured food reintroduction phase was a stepped transition to a food-based diet (about 50% carbohydrate, 35% total fat, and 15% protein), which was tailored as per individual preference in discussion with the dietitian. The liquid-based diet was tapered gradually within 2 to 8 weeks, before switching to full food-based weight loss maintenance. The participants were reviewed on a fortnightly basis in this phase by a member of the clinical team.

#### 2.3.3. Phase 3: Weight Loss Maintenance (Weeks 19-52)

The weight loss maintenance phase included a food-based diet, provided with an individually tailored energy prescription by the dietitian. The participants were reviewed at least once a month until the completion of the program at 52 weeks by a member of the clinical team. In case of relapse defined as a weight regain of >4 kg as described in the DiRECT protocol [25], a 4-week total diet replacement followed by a 2–4-week food reintroduction was offered.

### 2.4. Data Collection

Data were collected at three timepoints by a researcher who was not part of the clinical team: at the start of the program (baseline), after the completion of the total diet replacement (12 weeks), and after the completion of the structured diabetes program (52 weeks). The data collection measures at each timepoint are summarised in Table 1.

Data on costs associated with the implementation of DiRECT-Australia were collected over the study duration, including intervention set-up cost, intervention running cost, and general practice and dietitian visit/consultation cost. The intervention cost per participant over 52 weeks of the DiRECT intervention was calculated based on the methodology adopted by the DiRECT UK study [27]. The intervention set-up and running cost relating to the general practice and practice nurse were calculated using the Australian Medicare Benefits Schedule and Nurses Award 2020. All the costs were converted and presented in 2022 Australian dollars [28].

The low-calorie meal replacement soups and shakes used in this study were provided to the participants at a subsidised cost of A$2 per sachet. With four sachets consumed per day, the total cost was A$112 per fortnight. In comparison, meal replacement products are commercially available in Australian supermarkets, chemists, and pharmacies at comparatively higher costs. For example, Optifast products (Nestlé, Vaud, Switzerland) cost around A$45-A$65 per 12-18 sachets [29]. Participants were asked a question at baseline related to the willingness to pay for the meal replacements: “What is the most you would be prepared to pay per fortnight for your low-calorie shake?”

### 2.5. Outcomes

The primary outcome of this study was the proportion of participants achieving diabetes remission, defined as an HbA1c < 6.5% (<48 mmol/mol) off all diabetes therapies for at least three months [20], from baseline to 52 weeks. Secondary outcomes included changes in medications, weight loss, and improvement in glycaemic control as measured by reduction in HbA1c, at 12 weeks and 52 weeks. Other outcomes included improvements in clinical health outcomes and biochemical profile, including blood pressure, lipid profile, kidney and liver function, and serum-based liver fibrosis scores. Outcomes also included changes in self-reported physical activity, readiness to change lifestyle behaviour, and quality of life and wellbeing using measures described in Table 1, along with intervention cost and feasibility and fidelity of the diabetes remission service.

### 2.6. Statistical Analysis

The Statistical Package for Social Sciences, Version 29 (SPSS for macOS; SPSS Inc., Chicago, IL, USA) was used for the statistical analysis. Continuous variables were presented as mean and 95% Confidence Intervals (95% CI), and categorical variables were presented using percentage and frequencies. A Shapiro–Wilk test was performed to assess the normality of the distribution of the data. Normally distributed continuous data were compared using the paired *t*-test, whereas nonparametric data were compared using the Wilcoxon signed-rank test. Multiple imputations with five imputations were performed for missing data. The imputation or predictive model used linear regression which included the following variables: age, gender, diabetes duration, number of diabetes medications, and HbA1c at baseline; pooled results were presented. Mean differences in observations at two timepoints were calculated as an effect size by subtracting the baseline value from the follow-up value. Categorical variables were compared using McNemar's test. All tests with *p* values < 0.05 were considered statistically significant after adjusting for multiple testing using the Benjamini–Hochberg method with a false discovery rate of 0.1. The EQ-5D health state utility was calculated by assigning values to different levels of the EQ-5D dimensions, summing them up, and normalising the sum by dividing it by the maximum possible value using a value set specific to the Australian population [36, 37]. For cost analysis, mean cost was calculated by averaging resource cost per participant.

### 2.7. Ethics Approval

Ethics approval was granted by the Western Sydney University Human Research Ethics Committee (reference number H13427). This study was conducted in accordance with the Declaration of Helsinki, and written informed consent was obtained from all participants in the study.

## 3. Results

**Figure 1:**
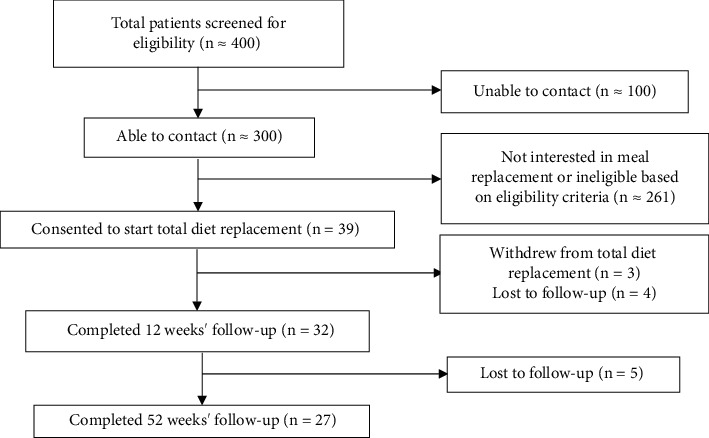
Flowchart of the participant recruitment and program completion.

A total of 39 participants were recruited in this study (≈13% of total patients contacted), of whom 32 (82.1%) completed the 12-week total diet replacement and 27 (69.2%) completed 52 weeks in the structured diabetes remission program as shown in Figure 1. Participant characteristics are shown in Table 2.

### 3.1. Weight Loss and Glycaemic Control

Table 3 shows the statistically significant weight decrease of -12.0 kg (95% CI: -9.6, -14.4; *p* < 0.001) at 12 weeks, followed by weight increase during the food reintroduction phase and weight loss maintenance phase by 2.9 kg (95% CI: 6.2, -0.3; *p* = 0.074). Compared to baseline, there was a statistically significant weight decrease of -9.1 kg (95% CI: -5.2, -12.9; *p* < 0.001) at 52 weeks. Similarly, HbA1c decreased by -1.1% at 12 weeks (95% CI: -1.6, -0.6; *p* < 0.001) and by -0.6% at 52 weeks (95% CI: -1.1, -0.1; *p* = 0.013), as compared to baseline.

### 3.2. Diabetes Remission and Changes in Medications

At the start of the program, 23.1% of participants were only on diet/lifestyle modification, with the remaining on noninsulin antihyperglycaemic agents. The most common medications prescribed at baseline are shown in Table 2. Compared to baseline, there was a statistically significant reduction in medication load at 12 weeks, with only 6.3% of participants on diabetes medication (all on metformin monotherapy). Similarly, the proportion of participants on diabetes medication at 52 weeks (37.0%) was statistically significantly lower compared to baseline (*p* = 0.006). At the end of 12 and 52 weeks, 93.8% (30/32) and 55.6% (15/27) of participants with follow-up data met the criteria for diabetes remission, respectively.

Antihypertensive agent use dropped from 41.0% at baseline to 12.5% (*p* = 0.012) at 12 weeks. By 52 weeks, antihypertensive agent use was back to baseline (59.3%, *p* = 0.999) with no difference in the proportion of people using monotherapy or combination antihypertensive agents across each timepoint, and no significant change in measured systolic or diastolic blood pressure. There was no change in cholesterol medication use at baseline compared to 12 or 52 weeks.

### 3.3. Changes in Biochemical Profile

Table 3 shows reductions in triglycerides over 52 weeks. While Gamma-glutamyl Transferase (GGT) was lower at 12 and 52 weeks, Alanine Transaminase (ALT), Aspartate Aminotransferase (AST), and the serum-based liver fibrosis scores remained unchanged.

### 3.4. Physical Activity and Readiness to Change Lifestyle Behaviour

Table 4 shows that more participants were in the action and maintenance stages of readiness to change diet at 12 weeks (except for fibre, protein, and vegetables), but none were significantly different at 52 weeks. However, more participants were in the action and maintenance stages of readiness to change weight and physical activity at both 12 and 52 weeks (except for reducing sedentary behaviour at 52 weeks). Despite this readiness to change, no change in questionnaire-derived physical activity measures was observed.

### 3.5. Quality of Life and Wellbeing and Intervention Cost

As shown in Table 4, the quality of life and wellbeing measures (EQ-5D health state utility, EQ-5D VAS, and WHO-5 Well-Being Index) increased (*p* < 0.001) over the course of 12 weeks, and their change remained significant at 52 weeks. The breakdown of the intervention resource use and cost are shown in Table 5. The average intervention cost in the DiRECT-Australia T2DM Remission Service over 52 weeks was A$2912 per participant and A$5242 per remission.

### 3.6. Feasibility and Fidelity of the Service

Overall, 10 general practices were involved in the delivery of the DiRECT-Australia intervention. The general practice size ranged from a solo practitioner to large practices with 14 practitioners, and all practices had one or more practice nurses, with no association between the number of patients recruited and the practice size. The average value that participants were prepared to pay for the low-calorie shakes was A$92.5 (95% CI: A$75.8, A$109.3) per fortnight. All 27 participants completing 52 weeks in the program agreed that the cost of the meal replacements was reasonable, and they were willing to pay if the service was provided on a long-term basis. No adverse health-related events were reported during the 52-week study duration.

## 4. Discussion

This evaluation demonstrated that a structured and evidence-based T2DM remission service developed in the UK can be successfully replicated in an Australian primary care setting. High rates of remission of diabetes were achieved at 12 and 52 weeks (93.8% and 55.6%, respectively) among patients with recent onset T2DM. This was accompanied by significant decrease in weight by -12.0 kg at 12 weeks and -9.1 kg at 52 weeks, along with significant improvements in lipids, GGT, quality of life, and wellbeing measures over 52 weeks. These findings, together with a substantial willingness to pay for diet replacement products among participants, and 82.1% and 69.2% of those agreeing to participate completing 12 and 52 weeks in the program, respectively, support the feasibility and effectiveness of a structured diabetes remission service in the Australian setting.

Corroborating the findings of the DiRECT UK study [17], this study demonstrated that T2DM is not necessarily a permanent and progressive condition, and diabetes remission is achievable in many patients with recently diagnosed T2DM, aided by a loss of abnormal ectopic fat accumulation in the liver and pancreas [18, 19, 39]. However, diabetes remission without an intervention is rare, especially within a community setting among a population receiving standard clinical care [40]. This study was successful in adding to the evidence that diabetes remission is achievable through an intensive weight loss intervention delivered in a primary care setting. The proportion of participants in remission at the end of 52 weeks of DiRECT-Australia (55.6%) was in the range of other studies including the DiRECT UK study (46%) [17] and the DIADEM-I study conducted in a primary care setting in Qatar (61%) [41]. While two-year outcomes were not collected in this study, promising results were observed in the DiRECT UK study with more than a third of the participants (36%) achieving sustained remission at the end of two years [23]. This was also observed in a recent study among patients with T2DM attending a single general practice in Sydney, where diabetes remission was achieved in a notable proportion of participants at 12 and 24 months (11.8% and 32.4%, respectively) using a three-month partial meal replacement plan [11]. Moreover, a recent study demonstrated that people with BMI < 27 kg/m^2^, which represents 1 in 6 people of white European ethnicity presenting with T2DM, can also achieve remission by a more modest weight loss [39]. These findings highlight the potential of dietary weight loss in achieving remission in individuals with T2DM, irrespective of their BMI.

The decrease in weight at 52 weeks (-9.1 kg) in this study was comparable with that in the DiRECT UK study (-10.0 kg) [17] as well as the DIADEM-I study (-11.98 kg) [41], which demonstrates the effectiveness of a structured and intensive weight loss intervention in achieving clinically meaningful weight loss. This was further demonstrated by the Look AHEAD (Action for Health in Diabetes) study, where participants achieved similar weight change at 52 weeks (-8.6 kg) through an intensive weight loss intervention combining diet and physical activity [42]. The mean BMI of the participants in this study was higher than that in the DiRECT UK study (37 and 35.1 kg/m^2^, respectively). This is because the DiRECT UK study only included participants with BMI of 27–45 kg/m^2^, while this study included all participants with BMI ≥ 27 kg/m^2^. Despite the challenges that could be posed by high BMI in long-term weight loss maintenance [43], participants were able to achieve significant weight loss at both 12 and 52 weeks (12 kg and 9.1 kg, respectively), with weight regain of only 2.9 kg during the food reintroduction phase and weight maintenance phase. In the DiRECT UK study, significant weight loss of 14.5 kg was achieved at 12 weeks, with comparable weight regain during the food reintroduction phase and weight loss maintenance phase (1 kg and 1.9 kg, respectively).

The significant weight loss in this study was also accompanied by improved glycaemic control and reduced diabetes medication load, which has also been noted by previous studies among patients with T2DM [11, 44, 45]. The decrease in HbA1c at 52 weeks (-0.6%) was comparable with that in the DiRECT UK study (-0.9%), with a similar proportion of participants still on diabetes medication by the end of 52 weeks (37% and 26%, respectively) [17]. While weight loss and reduction in HbA1c were significantly lower at both 12 and 52 weeks compared to baseline, there was a trend towards a slight increment in weight and HbA1c from 12 to 52 weeks, which may suggest the need for additional support in the weight maintenance phase. However, these results are even more impressive given that patients within local outpatient clinics and in primary care often do not achieve glycaemic targets in spite of specialist input and improvements in weight and glycaemia [46–48].

The implementation of the DiRECT intervention also led to improvements in clinical health outcomes, including a reduction in triglycerides at 12 and 52 weeks, similar to the DiRECT UK study [17]. As for liver function parameters, GGT values were lower at 12 and 52 weeks, with no significant changes in ALT, AST, or serum-based liver fibrosis scores. These results can be linked with the findings of a recent study which demonstrated that weight-loss-induced diabetes remission is associated with a major fall in liver fat export and decreased liver-derived triglyceride and intrapancreatic fat [49]. It has been previously noted that GGT may be a more sensitive detector of Nonalcoholic Fatty Liver Disease (NAFLD) than ALT and could be added to first-time liver function tests to increase detection of liver disease [50]. GGT may also help identify individuals with high risk of NAFLD as elevated GGT levels have been shown to be associated with incidence of NAFLD even before an ultrasound diagnosis [51, 52]. Evidence also suggests that weight loss can lead to improvements in liver function tests and serum-based liver fibrosis scores especially in patients with NAFLD [53–55]. Nonetheless, larger trials of a longer duration are needed to confirm this effect among patients with recent onset T2DM.

The DiRECT intervention led to significant changes in the participants' readiness to change lifestyle behaviour, where more participants were in action and maintenance stages of readiness to change diet at 12 weeks, while readiness to change weight and physical activity were maintained at both 12 and 52 weeks. In spite of the DiRECT intervention being a diet-based intervention, the finding that only readiness to change weight and physical activity were maintained over 52 weeks suggests the need to further motivate participants for dietary change. Nevertheless, the self-reported quality of life and perceived wellbeing increased and remained significant at both 12 and 52 weeks, which reflect the effectiveness of a structured diabetes program in achieving broader benefits for the participants. This finding was also reported in the DiRECT UK study, where quality of life was also measured using EQ-5D, and significant improvement was observed at 52 weeks [17]. Overall, the implementation of the DiRECT intervention resulted in diabetes remission and wide-ranging associated benefits at a total intervention cost of A$2912 per participant. Comparable one-year programme costs of £1137 per participant (approximately A$2499 in 2022 Australian dollars) were reported for the DiRECT UK study in 2019 [27]. Nonetheless, detailed and longer duration health economics studies are needed, particularly by modelling the HbA1c and diabetes remission projection, to estimate the cost effectiveness of the DiRECT intervention, as the intervention set-up costs would be lower per patient as the number of people joining the program increases.

This study was conducted in a real-life setting, with participants recruited by general practices, and approximately 13% of the total patients contacted consented to start the total diet replacement. It is plausible that the supporting information provided to the patients, in terms of the success of the DiRECT UK study as well as the DiRECT intervention being based on the understanding of reversibility of T2DM, may have piqued the interest of potential participants [17, 18, 23]. While high dropout rates are often reported for interventions delivered in a primary care setting [11, 56], the acceptability of DiRECT-Australia was demonstrated by the high proportion of participants (82.1% and 69.2%, respectively) being able to complete the 12-week total diet replacement phase and 52 weeks of the structured diabetes remission program. This was further confirmed by a substantial willingness to pay for diet replacement products among participants. The finding that the DiRECT intervention can be successfully run in an Australian primary care setting, regardless of the general practice size, supports the feasibility of a structured diabetes remission service in the Australian setting. A similar diabetes remission service is currently being delivered in certain areas of England and Scotland by the National Health Service (NHS) and provides a low-calorie total diet replacement treatment to eligible individuals living with overweight and T2DM [57]. This study provides the first Australian evidence on the effectiveness of such a diabetes remission service including participants' willingness to pay for meal replacements. As recommended in the position statement by Diabetes Australia on T2DM remission, this research provides a better understanding of the real-world implementation of a T2DM remission program [8]. This study has important implications for practice, including the possibility of changing the current treatment paradigm by going in early and treating recently diagnosed T2DM with total dietary replacement to reverse the effects of T2DM and with the aim of achieving diabetes remission. This is in line with the Australian National Diabetes Strategy 2021–2030 [58], which suggests offering intensive dietary interventions to people with T2DM who are aiming for remission. A wider implementation of the diabetes remission service across Australia would not only reduce the burden on specialist clinics [47] but ensure that anyone with recent onset T2DM is offered the best possible chance to achieve remission.

The study findings should be interpreted considering some limitations. This was a pilot prospective evaluation study of a T2DM remission service and did not include a control group or randomisation. However, a randomised controlled trial design may no longer be required given the prior evidence on the effectiveness of intensive dietary interventions in achieving diabetes remission [17, 23, 41]. Nonetheless, participants in this study were recruited following detailed eligibility criteria as in most clinical trials, and the intervention in this study was structured and evidence-based with frequent follow-up of participants [25]. Another limitation is that the generalisability of the study findings is limited by the small sample (*n* = 39) of participants recruited by the general practices in a region of Sydney. The challenges in recruiting patients could be associated with the difficulty in supporting and motivating patients to lose weight in a primary care setting, as previously noted [11, 59]. In addition, these challenges were amplified by the recruitment and intervention period spanning the 2019-20 Australian bushfire season and the challenging times of the coronavirus disease 2019 (COVID-19) pandemic, which required some of the follow-up to be conducted remotely instead of face-to-face. Nonetheless, as this study was conducted under real-life primary care conditions, the participants were typical of patients recently diagnosed with T2DM who are routinely managed in this setting. Given the multiethnic profile of the study population, the study findings may be applicable to people from diverse ethnic backgrounds residing in Australia.

## 5. Conclusions

This evaluation study of DiRECT-Australia demonstrated that translation of a structured and evidence-based T2DM remission service developed in the UK to an Australian primary care setting resulted in diabetes remission in many patients with recent onset T2DM. This was accompanied by significant weight loss and improvement in glycaemic control, along with broader benefits such as improved self-reported quality of life and perceived wellbeing. These results are consistent with the DiRECT UK findings and suggest a substantial willingness to pay for diet replacement products among participants and support the feasibility of a structured diabetes remission service in the Australian setting.

## Figures and Tables

**Table 1:** Summary of data collection measures at different timepoints during the study.

Data collection measures	Baseline	12 weeks	52 weeks
Demographics (e.g., age, gender, education, and ethnic background)	√		
Past medical history including diabetes complications, comorbidities, and presence of cardiometabolic risk factors	√		
Weight, blood pressure, blood glucose level, and body mass index (BMI)	√	√	√
Medications (includes names, dose, and frequency of all medications currently prescribed)	√	√	√
Blood test (HbA1c, kidney and liver function, urine albumin-to-creatinine ratio, serum lipids, and full blood count)	√	√	√
Serum-based liver fibrosis scores (Fibrosis-4 (FIB-4) Index for Liver Fibrosis [30], AST to Platelet Ratio Index (APRI) [31], and Nonalcoholic Fatty Liver Disease (NAFLD) fibrosis score [32])	√	√	√
International Physical Activity Questionnaire (IPAQ) short form [33]	√	√	√
Readiness to change lifestyle behaviour using validated questionnaire based on the transtheoretical (“stages of change”) questionnaire which includes readiness to change diet (7 items), physical activity (5 items), and weight (1 item) [34, 35]	√	√	√
Quality of life using EuroQol Five-Dimension (EQ-5D) Health State Utility and Visual Analog Scale (VAS) [36, 37]	√	√	√
Wellbeing using World Health Organization Five (WHO-5) Well-Being Index [38]	√	√	√
Feasibility and fidelity of the service	√		√

**Table 2:** Baseline characteristics of study participants.

Characteristics [mean (SD) or *n* (%)]	Participants (*n* = 39)
*Sociodemographic characteristics*	
Age (years)	50.9 (10.8)
Gender (female)	21 (53.8%)
In paid employment	29 (74.4%)
Country of birth (Australia)	28 (71.8%)^#^
*Comorbidities*	
Type 2 diabetes duration (years)	3.2 (1.7)
Hypertension	17 (43.6%)
Dyslipidaemia	19 (48.7%)
Obstructive sleep apnoea	8 (20.5%)

*Medications*	
Number of noninsulin antidiabetic medications	0: 9 (23.1%) 1: 18 (46.1%) ≥2: 12 (30.8%)
Noninsulin antidiabetic medications prescribed (*n* = 30)	Metformin: 28 (93.3%) SGLT-2 inhibitors: 8 (20.7%) DPP-4 inhibitors: 4 (13.3%) Sulphonylureas: 2 (6.6%) GLP-1 receptor agonists: 1 (3.3%)
Number of antihypertensive medications (*n* = 17)	0: 1 (5.9%) 1: 6 (35.3%) ≥2: 10 (58.8%)
Lipid-lowering medication prescribed	21 (53.9%)

*Behavioural factors*	
Consuming any alcohol	12 (30.8%)^##^
Smoking status	Current smoker: 1 (2.6%) Ex-smoker: 11 (28.2%)

Abbreviations: DPP-4: dipeptidyl peptidase-4; GLP-1: glucagon-like peptide-1; SD: standard deviation; SGLT-2: sodium-glucose cotransporter-2. ^#^Remaining participants were born in Europe, East Asia, Southeast Asia, South Asia, and Latin America. ^##^All the participants reported an alcohol consumption consisting of 3 or less standard drinks per week (≤5.25 alcohol units/week).

**Table 3:** Changes in anthropometric, clinical, and biochemical measures at 12 and 52 weeks' follow-up (*n* = 39).

Variable Mean (CI) or *n* (%)	Baseline	12 weeks follow-up		52 weeks follow-up	
		Mean difference^#^	*p* value	Mean difference^#^	*p* value
Weight (in kg)	104.6 (98.5, 110.8)	-12.0 (-9.6, -14.4)	<0.001^∗^	-9.1 (-5.2, -12.9)	<0.001^∗^
BMI (in kg/m^2^)	37.0 (35.3, 38.7)	-4.0 (-3.2, -4.9)	<0.001^∗^	-2.5 (-1.4, -3.5)	<0.001^∗^
Waist circumference (in cm)	114.9 (108.9, 120.9)	-14.1 (-8.4, -19.8)	<0.001^∗^	-8.3 (-3.6, -13.0)	<0.001^∗^
SBP (in mmHg)	129.7 (125.1, 134.4)	-2.7 (2.4, -7.8)	0.295	-0.8 (3.9, -5.4)	0.727
DBP (in mmHg)	83.9 (81.1, 86.6)	-3.0 (0.3, -6.3)	0.071	-2.4 (0.8, -5.5)	0.128
Capillary blood glucose (in mmol/L)	8.4 (7.0, 9.7)	-1.8 (-0.5, -3.2)	0.007^∗^	-1.6 (-0.5, -2.7)	0.004^∗^
*Biochemical profile*					
Measure of blood glucose					
HbA1c (in %)	7.4 (7.0, 7.8)	-1.1 (-0.6, -1.6)	<0.001^∗^	-0.6 (-0.1, -1.1)	0.013^∗^
Kidney function test					
Urea (in mmol/L)	6.9 (3.7, 10.1)	-1.1 (2.0, -4.2)	0.473	-0.8 (2.3, -3.9)	0.584
Creatinine (in *μ*mol/L)	72.6 (65.9, 74.2)	-1.4 (3.2, -6.0)	0.536	5.9 (11.6, -0.3)	0.051
eGFR (in ml/min/1.73 m^2^)	85.7 (82.8, 88.5)	-0.6 (-2.7, 1.5)	0.694	-1.5 (2.1, -5.2)	0.363
Urine ACR (in mg/mmol)	3.1 (1.5, 4.8)	0.8 (2.8, -1.2)	0.465	2.5 (8.0, -3.1)	0.346
Lipid profile					
Total cholesterol (in mmol/L)	4.7 (4.3, 5.0)	-0.3 (0.1, -0.7)	0.105	-0.2 (0.2, -0.5)	0.117
Triglyceride (in mmol/L)	2.7 (1.9, 3.5)	-0.9 (-0.2, -1.7)	0.013^∗^	-0.6 (-0.3, -0.9)	<0.001^∗^
HDL-cholesterol (in mmol/L)	1.2 (1.1, 1.3)	0.0 (0.1, -0.1)	0.431	0.1 (0.2, 0.1)	0.004^∗^
LDL-cholesterol (in mmol/L)	2.5 (2.2, 2.8)	-0.3 (0.1, -0.7)	0.120	-0.1 (0.3, -0.5)	0.562
Liver function test					
Total bilirubin (in *μ*mol/L)	11.7 (9.9, 13.6)	0.8 (2.1, -0.5)	0.273	0.9 (2.9, -1.0)	0.377
Albumin (in g/L)	43.0 (42.0, 43.9)	0.2 (1.5, -1.0)	0.791	0.9 (3.1, -1.3)	0.440
ALP (in IU/L)	78.4 (69.7, 87.2)	0.7 (7.2, -5.8)	0.839	-1.5 (6.8, -9.8)	0.715
GGT (in IU/L)	50.6 (39.2, 61.9)	-10.5 (-2.0, -19.0)	0.015^∗^	-12.0 (-0.3, -23.8)	0.044
ALT (in IU/L)	47.1 (34.5, 59.6)	-7.2 (5.7, -20.0)	0.270	0.8 (25.1, -23.5)	0.953
AST (in IU/L)	32.5 (25.7, 39.4)	-2.9 (3.8, -9.6)	0.392	3.9 (18.9, -11.1)	0.612
Serum-based liver fibrosis score					
FIB-4 index	1.1 (0.9, 1.3)	0.1 (0.3, -0.2)	0.882	0.2 (0.5, -0.2)	0.432
APRI score	0.4 (0.2, 0.5)	-0.1 (0.1, -0.2)	0.109	0.2 (0.4, -0.1)	0.491
NAFLD fibrosis score	-0.6 (-1.2, -0.1)	0.1 (0.8, -0.6)	0.891	0.1 (0.6, -0.3)	0.720

Abbreviations: ALP: alkaline phosphatase; ALT: alanine transaminase; APRI: AST to platelet ratio index; AST: aspartate aminotransferase; BMI: body mass index; CI: confidence interval; DBP: diastolic blood pressure; eGFR: estimated glomerular filtration rate; FIB-4: fibrosis-4 index for liver fibrosis; GGT: gamma-glutamyl transferase; HbA1c: glycated haemoglobin; HDL: high-density lipoprotein; LDL: low-density lipoprotein; NAFLD: nonalcoholic fatty liver disease; SBP: systolic blood pressure; urine ACR: urine albumin-to-creatinine ratio. ^#^Mean difference = follow-up value-baseline value. Note: *p* values presented are unadjusted for multiple testing; *p* values marked with an “∗” retained significance after Benjamini-Hochberg correction for multiple testing. eGFR was calculated using the Chronic Kidney Disease Epidemiology Collaboration (CKD-EPI) equation.

**Table 4:** Changes in physical activity, readiness to change lifestyle behaviour, and quality of life and wellbeing at 12 and 52 weeks' follow-up.

Variable *n* (%) or mean	Baseline (*n* = 39)	12 weeks follow-up^∗^		52 weeks follow-up^∗^	
		Follow-up	*p* value	Follow-up	*p* value
*Physical activity IPAQ-short form 7 days*					
Have you participated in vigorous activity in the past 7 days	15 (38.5%)	10 (40.0%)	0.599	11 (50.0%)	0.687
Have you participated in moderate activity in the past 7 days	19 (48.7%)	16 (64.0%)	0.357	13 (59.1%)	1.000
Have you walked more than 10 min at a time in the last 7 days	34 (87.2%)	25 (100%)	1.000	19 (86.4%)	0.500
IPAQ: MET-min per week	2820.3	2782.3	0.981	2740.9	0.998
*Readiness to change lifestyle behaviour (nand % for action and maintenance stage)*					
Dietary change: readiness to change diet					
Do you drink water and other nonsugary drinks instead of sugary drinks/fruit juice	30 (76.9%)	24 (96.0%)	0.031	21 (95.5%)	0.625
Do you eat at least four or more servings of vegetables daily	21 (53.8%)	15 (60.0%)	0.754	14 (63.6%)	1.000
Do you eat at least three different protein foods every 1-2 days	30 (76.9%)	17 (68.0%)	0.508	17 (77.3%)	1.000
Do you eat less fat overall?	14 (35.9)	23 (92.0%)	<0.001	15 (68.2%)	0.125
Have you reduced amount of food you eat at each sitting	21 (53.8%)	24 (96.0%)	<0.001	16 (72.7%)	0.754
Do you eat more foods with fibre	23 (59.0%)	16 (61.0%)	0.999	18 (81.8%)	0.375
Do you eat less sugary foods and carbohydrates	24 (61.5%)	25 (100%)	0.004	17 (77.3%)	1.000
Physical activity: readiness to change physical activity					
Are you making yourself stronger?	10 (25.6%)	21 (81.4%)	<0.001	15 (68.2%)	0.004
Do you plan more activity in your day?	9 (23.1%)	23 (92.0%)	<0.001	14 (63.6%)	0.002
Do you plan more activity on weekends?	9 (23.1%)	21 (81.4%)	<0.001	14 (63.6%)	0.012
Have you increased the number of steps you take each day?	10 (25.6%)	22 (88.0%)	<0.001	14 (63.6%)	0.002
Have you reduced the amount of time you spend sitting?	15 (38.5%)	17 (68.0%)	0.008	10 (45.5%)	0.999
Weight: readiness to change weight					
Are you trying to reach your best weight	16 (41.0%)	23 (92.0%)	<0.001	19 (86.4%)	0.002
*Quality of life and wellbeing*					
EQ-5D health state utility	0.80	0.86	<0.001	0.87	<0.001
EQ-5D VAS	47.5	63.6	<0.001	64.2	<0.001
WHO-5 Well-Being Index	55.7	68.8	<0.001	65.7	<0.001

Abbreviations: CI: Confidence Interval; EQ-5D: EuroQol Five-Dimensions; IPAQ: International Physical Activity Questionnaire; MET: Metabolic Equivalent of Task; VAS: Visual Analog Scale; WHO: World Health Organization. ^∗^12 weeks survey was completed by 25 participants and 52 weeks survey was completed by 22 participants. All the statistically significant *p* values retained significance after Benjamini-Hochberg correction for multiple testing.

**Table 5:** Intervention resource use and cost per participant (*n* = 39) over 52 weeks of the DiRECT intervention.

Intervention cost component	Quantity	Unit cost (A$)	Total cost (A$)	Cost per participant
*Intervention set-up cost*				
Counterweight-Plus training^a^	4 practices	550	2200	56.4
Practice nurse time^b^	16 h/practice	41.4	2649.6	67.9
Total intervention set-up cost			4740.8	124.3
*Intervention running cost*				
12 weeks of total diet replacement phase	336 sachets per participant	4.0	52416	1344.0
8 weeks of food reintroduction phase	112 sachets per participant	4.0	17472	448.0
Counterweight-Plus booklets	39	40	1560	40.0
Total intervention running cost			71448	1832.0
*General practice and dietitian visit/consultation*				
GP visit (GP and/or practice nurse) (per participant)^c^	12	39.8	18626.4	477.6
Dietitian	10 hours per participant	47.8	18704.4	478.0
Total cost of GP and dietitian consultation			37330.8	955.6
Total intervention cost per participant				2911.9
Total intervention cost per remission				5241.5

^a^Cost of unit of counterweight intervention is per Counterweight UK. ^b^Based on *Nurses Award 2020* (rates were calculated based on registered nurse Level 5 Grade 1). ^c^Based on Australian Medicare Benefits Schedule Item 23; Group A1; Subheading 2-Level B.

## Data Availability

The data used to support the findings of this study are available from the corresponding author upon reasonable request.
